# Systemic inflammation and chronic kidney disease in a patient due to the *RNASEH2B* defect

**DOI:** 10.1186/s12969-021-00497-2

**Published:** 2021-01-22

**Authors:** Tingyan He, Yu Xia, Jun Yang

**Affiliations:** Department of Rheumatology and Immunology, Shenzhen Children’s Hospital, 7019 Yitian Road, Shenzhen, 518038 China

**Keywords:** Auto-inflammation, Autoimmunity, Aicardi-Goutieres syndrome, Chronic kidney disease, *RNASEH2B*

## Abstract

**Introduction:**

Aicardi-Goutières (AGS) is a rare immune dysregulated disease due to mutations in *TREX1, RNASEH2A, RNASEH2B, RNASEH2C, SAMHD1, ADAR1,* or *IFIH1.* Clinical features include basal ganglia calcifications, white matter abnormalities, and cerebral atrophy. Severe systemic inflammation and chronic kidney disease (CKD) are extremely rare in AGS. Herein, we report a patient presenting with systemic inflammation and CKD to broaden the clinical phenotype spectrum of the *RNASEH2B* defect.

**Methods:**

All testing and molecular genetic analysis were performed after obtaining the informed consent of the parents. Demographic, clinical, and laboratory findings were abstracted from outpatient and inpatient encounters. Cerebral magnetic resonance imaging (MRI), computed tomography (CT) scans, and renal biopsy histopathology reports were reviewed and summarized. Whole exome sequencing (WES) was performed on peripheral blood cells. After exposure to cGAMP in vitro for 24 h, mRNA expression of 12 IFN-stimulated cytokine genes in PBMCs was assessed. Serum cytokine levels were detected by Milliplex.

**Results:**

A 11-year-old girl presented with recurrent aseptic fever, arthritis, chilblains, failure to thrive, mild hearing loss, and neurological manifestations. Laboratory and immunologic findings demonstrated lymphopenia, low complement levels, positive autoantibodies, elevated levels of acute-phase reactants and inflammatory cytokines. Cerebral imaging showed cerebral atrophy, white matter abnormalities, and intracranial calcification. Renal biopsy showed glomerular sclerosis in 3 of 14 glomeruli, infiltration of lymphocytes and other mononuclear cells. WES revealed a homozygous and heterozygous mutations in *RNASEH2B*. Over-expression of IFN-stimulated cytokine genes was observed, including IFI44, IFI27, IFIT1, IFIT2, IFIT3, ISG15, OAS1, and SIGLEC1.

**Conclusions:**

To date, only two cases with AGS have been reported to have renal disease. Here, we describe a patient with both homozygous and heterozygous variants in *RNASEH2B,* presenting with neurological manifestations, persistently systemic autoinflammation, and CKD. CKD has never been reported in patients with AGS due to the *RNASEH2B* defect*.*

**Trial registration:**

Not applicable; this was a retrospective study.

**Supplementary Information:**

The online version contains supplementary material available at 10.1186/s12969-021-00497-2.

## Background

Aicardi-Goutières (AGS) is a rare immune dysregulated disease due to mutations in *TREX1, RNASEH2A, RNASEH2B, RNASEH2C, SAMHD1, ADAR1* or *IFIH1,* characterized by encephalopathy, dystonia, basal ganglia calcifications, white matter abnormalities, and cerebral atrophy [1, 2]. Although most patients experienced severe neurological dysfunction within the first year of life, some patients presented with later onset of this disease with mild neurological manifestations and normal intellectual function. Systemic inflammation is not typically persistent. Renal dysfunction has been rarely described in AGS [2]. Here, we report a patient with both homozygous and heterozygous mutations in *RNASEH2B*, presenting with later onset recurrent sterile fever, arthritis, chilblains, failure to thrive, mild hearing loss, and neurological manifestations, which may broaden the clinical phenotype spectrum of the *RNASEH2B* defect.

## Materials and methods

### Subjects

This study was approved by the Ethics Committee of Shenzhen Children’s hospital. All human subjects (or their guardians) provided written informed consent. Clinical data of a patient with both homozygous and heterozygous variants in *RNASEH2B* was collected. Fifteen healthy volunteers were included as healthy controls (HCs). Venous blood (3 mL) was collected from each study subject.

### Whole exome sequencing (WES)

Genomic DNA was extracted from peripheral blood cells isolated from the patient and her parent. The exonic regions and flanking splicing or intronic junctions of the whole genome were captured and sequenced using an Illumina HiSeq 2000 sequencer conducted by MyGenostics (Beijing, China). The FASTQ files were mapped to the human reference genome (hg19). The functional effects of variants were predicted using three algorithms (PolyPhen-2, SIFT, and MutationTaster), and amino acid conservation among species was analyzed. Sanger sequencing was used to confirm pathogenic variants. The primers used to target human RNASEH2B included (forward: CAGGGATTTGAAGCTCTTTGG) and (reverse: TAGTGCTCTGTCCTGCACTGG).

### Cell culture

Peripheral blood mononuclear cells (PBMCs) were isolated by Ficoll-Paque PLUS (GE Healthcare) gradient density centrifugation and ACK lysis (Quality Biological). PBMCs were resuspended in complete RPMI (cRPMI) medium (Gibco, USA) containing 10% fetal bovine serum (BI, Israel), 2 mM glutamine, and penicillin-streptomycin (100 U/mL each; Sigma-Aldrich, USA). Cells at 1 × 10^6^/mL were exposed to cyclic guanosine monophosphate-adenosine monophosphate (cGAMP, CST#35573) at the concentration of 10 μg/ml.

### Real-time PCR

After exposure to cGAMP in vitro for 24 h, mRNA expression of 12 IFN-stimulated cytokine genes in PBMCs was assessed. Total RNA was extracted from PBMCs isolated from the patient and five HCs by RNA isolation kit (DP424, TIANGEN). cDNA was derived following the GoScript Reverse Transcription System kit(A5001, Promega). Quantitative reverse transcription PCR analysis was performed with the GoTaq qPCR Master Mix (A6002, Promega). Primers for PCR included were described in the supplementary material.

### Quantification of cytokine levels

Plasma samples were isolated from the patient and 15 HCs. Cerebrospinal fluid (CSF) sample was collected from the patient. Blood samples were collected in vacutainers containing sodium heparin. Plasma cytokine analyses were determined on a bead-based immunoassay (Milliplex, HCYTOMAG-60 K, Millipore, USA) according to the manufacturer’s protocol.

### Statistical analysis

Data were analyzed using an unpaired two-tailed Student t-test. All statistical analyses were conducted in GraphPad Prism 7 software (GraphPad Software, Inc., San Diego, CA).

## Results

### Clinical manifestations

**Fig. 1:**
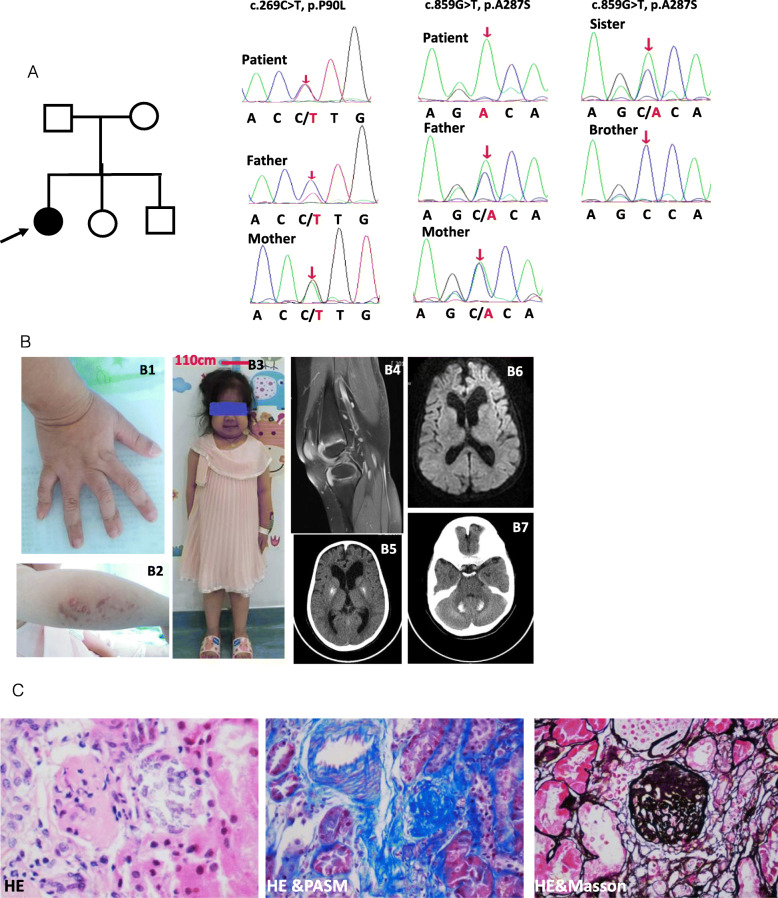
Clinical features and genetic analysis. **a** Pedigree and a homozygous and heterozygous variants in *RNASEH2B.* **b** Physical examinations and imaging findings showing deformed inter-phalangeal joints (B1), chilblains (B2), short statue (B3), synovial thickening(B4), cerebral atrophy and white matter abnormalities (B6), calcifications in basal ganglia and cerebellum (B5 and B7). **c** Renal biopsy findings (10 × 20) showing glomerular sclerosis, a mild proliferation of mesangial cells, infiltration of lymphocytes and other mononuclear cells

**Fig. 2:**
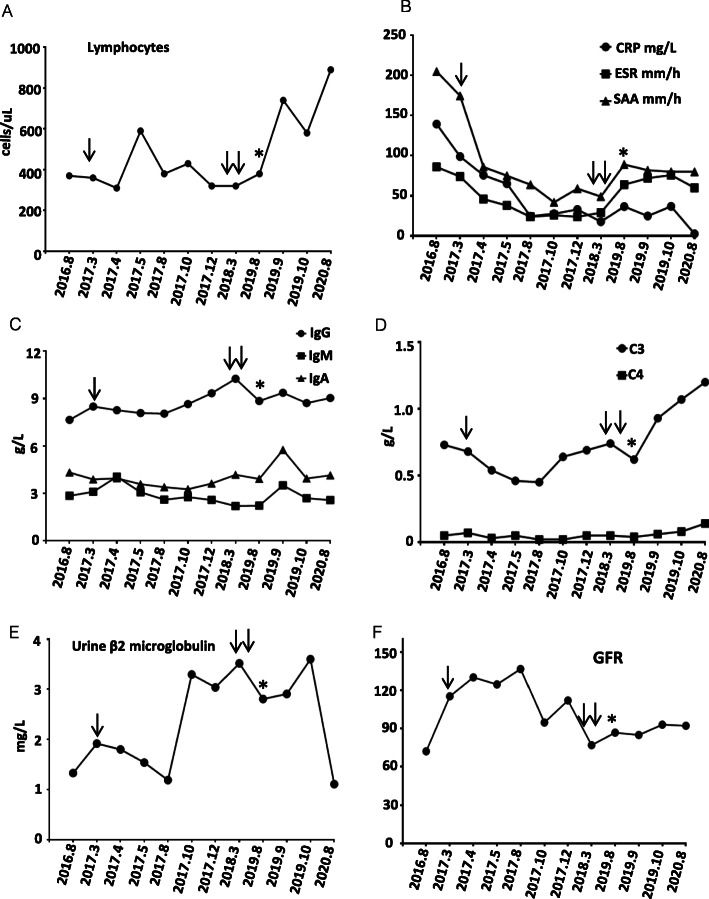
Abnormalities in laboratory findings. **a** lymphopenia. **b** Elevated CRP, ESR, and SAA levels. **c** Immunoglobulin levels showing intermediate elevated IgM and IgA levels. **d** Reduced C3 and C4 levels. **e** Persistently elevated levels of urine β2 macroglobulin. **f** Intermediate mild abnormalities in Cr and BUN. The normal reference ranges are as follows: lymphocytes (800 ~ 4000 cells/ul); CRP (0 ~ 10 mg/L); ESR (0 ~ 20 mm/h); SAA (0 ~ 6 mg/L); IgG (5.28 ~ 21 g/L); IgM (0.48 ~ 2.26 g/L); IgA (0.44 ~ 3.99 g/L); C3 (0.7 ~ 2.06 g/L); C4 (0.11 ~ 0.61 g/L); urine β2 macroglobulin (0 ~ 0.3 mg/L); GFR (80 ~ 120 ml/min). Single arrow and double arrows labeled the time when tocilizumab was started and discontinued, respectively. The asterisk labeled the time when tofacitinib was started

The patient presented with recurrent fever, arthritis, movement limitation, and growth retardation at the age of 11 years. At the age of 2 years, she began to suffer from recurrent aseptic fever with an intermittent resolution by traditional Chinese medicine. At the age of 5 years, she began to present with arthritis accompanied by mild hearing loss. She was born to a non-consanguineous healthy parent. At birth, her weight was 3 Kg, crown to heel length was 49 cm, and head circumference was 34 cm. She had standard motor and language development. Manifestations of failure to thrive had been significant since she was 3 years old. Physical examination revealed short stature with 106 cm top(<−3SD)(Fig. 1B3), macrocephaly with 54 cm head width, chilblains on elbows and lower limbs (Fig. 1B2), swelling and deformation of inter-phalangeal and knee joints (Fig. 1B1). Her Intelligence Quotient (IQ) test value was 108. Her EPQJ, CBCL, Conners, and HAMA scale tests did not demonstrate any social and psychological problems. Knee magnetic resonance imaging (MRI) revealed a thickness of the synovial capsule without invasive bone destruction (Fig. 1B4). Cerebral MRI showed cerebral atrophy and white matter abnormalities (Fig. 1B6). Intracranial calcification was further identified at the basal ganglia and cerebellum by CT scanning (Fig. 1B5 and Fig. 1B7). Laboratory findings revealed hyper-inflammation and chronic kidney disease (Fig. 1c, Fig. 2b, and Fig. 2f). Screening tests for fungal, bacteria, and *Mycobacterium tuberculosis* infection were all negative. Pathology of the renal biopsy showed glomerular sclerosis in 3 of 14 glomeruli, a mild proliferation of mesangial cells without deposits of any amyloid, immunoglobulin or immune complex, expansion of the tubular lumen, partial tubular atrophy, mild tubular fibrosis, infiltration of lymphocytes and other mononuclear cells (Fig. 1c). Granule degeneration and calcium deposition were visible in renal tubules. Austin score index for the evaluation of activity and chronicity was two and three points, respectively.

### Abnormality in clinical and immunologic phenotype

Analysis of peripheral blood leukocyte revealed persistent lymphopenia (Fig. 2a). Except for rheumatoid factor (RF) and anti-cyclic peptide containing citrulline (anti-CCP), other auto-antibodies for mixed connective tissue disease were all negative, including anti-nuclear antibody (ANA), anti-neutrophil cytoplasmic antibody (ANCA), anti-SSA, anti-SSB, anti-dsDNA, anti-thyroglobulin, anti-thyroperoxidase, and anti-TSH receptor antibodies. Other abnormal clinical and immunologic phenotypes included intermediate elevation of IgM and IgA levels (Fig. 2c) and mild reduction of C3 and C4 levels (Fig. 2d).

### Both homozygous and heterozygous variants in *RNASEH2B*

Whole exon sequencing revealed three variants in the *RNASEH2B* gene (OMIM:610181). There was a single nucleotide homozygous variant, c.859G > T, p.A287S (Fig. 1a). Predicted values of SIFT, PolyPhen_2, Mutation Taster, and GERP++ were 0.235, 0.721, 1, and 6.06, suggesting tolerated, possibly damaging, and disease-causing effects, respectively. Both parents carried a heterozygous mutation at the same locus. Another single-nucleotide heterozygous variant, c.269C > T, p.P90L, was identified (Fig. 1a). The predicted values of SIFT, PolyPhen_2, Mutation Taster, and GERP++ were 0.002, 0.988,1, and 4.69, suggesting damaging, probably damaging, and disease-causing effects, respectively. This heterozygous variation was further confirmed by Sanger in both her parents. Both variations were in the conserved domains. Pathogenic variants were not identified in other genes related to autoinflammation, autoimmunity, or inherited renal disorders (Supplemental Table).

### Over-expression of IFN-stimulated cytokine genes

**Fig. 3:**
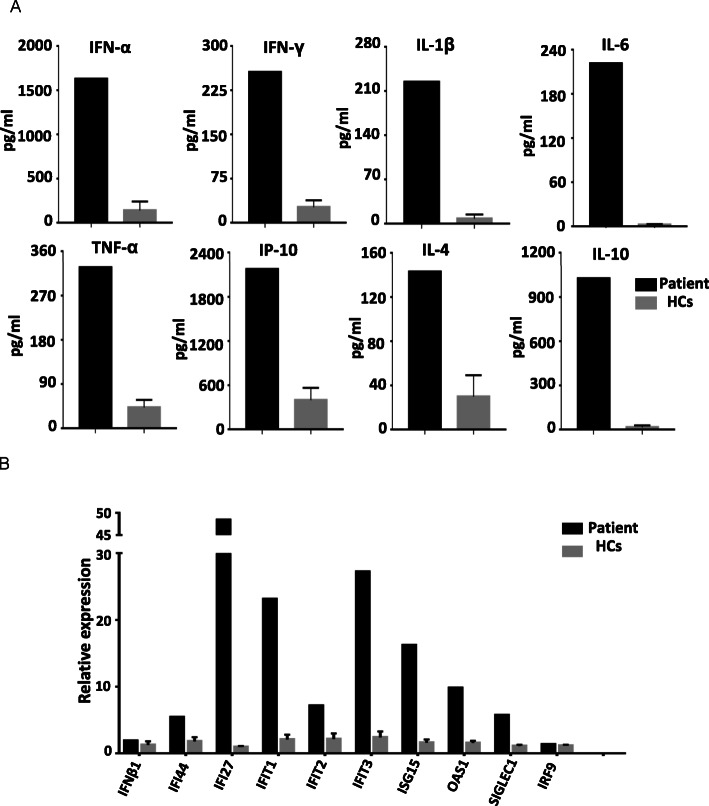
Abnormalities in plasma cytokines and IFN-stimulated cytokine genes. **a** Significantly elevated levels of all plasma cytokines in the patient. **b** Over-expression of IFN-stimulated cytokine genes in the patient, including IFI44, IFI27, IFIT1, IFIT2, IFIT3, ISG15, OAS1, and SIGLEC1. Normal mRNA expressions were observed in IFNβ1 and IRF9

After exposure to cGAMP in vitro for 24 h, mRNA expression of IFN-stimulated cytokine genes in PBMCs was detected by real-time PCR. In contrast to five healthy controls, over-expression of IFN-stimulated cytokine genes was observed in the patient, including IFI44, IFI27, IFIT1, IFIT2, IFIT3, ISG15, OAS1, and SIGLEC1. Normal mRNA expressions were found in IFNβ1 and IRF9 (Fig. 3b).

### Elevations in inflammatory cytokine levels

Compared to 15 age-matched healthy controls, plasma cytokine levels were significantly elevated, including interleukin (IL)- 1β, IL-6, tumor necrosis factor-α (TNFα), interferon-γ (IFN-γ), IFN-α, IL-4, IL-10, IL-12, IL-17A and IP-10 (Fig. 3a and Table 1). IFN-α level in CSF was very low.

**Table 1:** Other laboratory findings

Parameters	Before tocilizumab	12 weeks after tocilizumab	Before tofacitinib	48 weeks after tofacitinib	Reference Range
Rheumatoid factor (IU/ml)	879	810	2615	2660	0–20
Protein in CSF (mg/L)	541.9	NA	NA	NA	150–450
WBCs in CSF (10^6^/L)	1	NA	NA	NA	0–15
interferon-α in CSF (pg/ml)	22.1	NA	NA	NA	NA
interferon-α in plasma (pg/ml)	1627	1040	2309	1601.4	139.64 ± 96.54
IP10 in plasma (pg/ml)	2172	2160	3702	2135	397.43 ± 159.51
IFNγ in plasma (pg/ml)	453.6	260.8	850.5	282.9	26.32 ± 11.24
TNFa in plasma (pg/ml)	335.4	232.9	554.6	259.1	42.05 ± 14.76
IL1β in plasma (pg/ml)	224.15	208	274.3	218	7.75 ± 6.5
IL4 in plasma (pg/ml)	311	135.6	473	252	29.81 ± 18.48
IL6 in plasma (pg/ml)	221	142.6	230.3	156.2	5.5 ± 3.39
IL10 in plasma (pg/ml)	1025	540.9	731.4	568.6	14.36 ± 9.88
IL12 in plasma (pg/ml)	1507	1389	1372	1337	149.32 ± 139.9
IL17A in plasma (pg/ml)	64.9	44.8	43.7	48	15.55 ± 10.13
GM-CSF in plasma (pg/ml)	1146	1053	1067	1073.6	68.36 ± 44.74

### Treatment and outcome

A one-year course of growth hormone showed no response to improve her short stature. She had received long-term treatment of ibuprofen, methotrexate, folic acid, and prednisone for more than 5 years. Aseptic fever relapsed intermittently. Tocilizumab was started for the high dose dependence of glucocorticoids and elevated pro-inflammatory cytokine levels. Following a 48-week course of tocilizumab, the prednisone dose was gradually reduced to 0.2῀0.3 mg/Kg.d with partial improvement of some abnormal laboratory findings (Fig. 2b and Fig. 2f). However, urine β2 microglobulin level was persistently elevated markedly (Fig. 2e). Tocilizumab was discontinued. She began to receive tofacitinib (5 mg, twice every day) for the over-expression of IFN-stimulated cytokine genes. The 48-week course of tofacitinib led to partial response, including increased lymphocytes, C3 and C4 levels, reduced levels of urineβ2 microglobulin, C-reactive protein (CRP), and pro-inflammatory cytokines (Fig. 2a and Fig. 2d). However, chronic systemic inflammation was not completely controlled since the levels of inflammatory cytokines, erythrocyte sedimentation rate (ESR), and serum amyloid A (SAA) were still increased.

## Discussion

Biallelic mutations of *RNASEH2B* are most common in AGS. While three allelic variants in *RNASEH2B* have been identified in this patient. The population frequency of the variant c.859G > T, p.A287S in East Asian is 0.02, with two homozygotes demonstrated in ExAC Browser. This variation can cause reduced enzyme activity of RNase H2 and lower stability of the RNase H2 complexes, increasing susceptibility to systemic lupus erythematosus (SLE) [3]. The heterozygous variant c.269C > T, p.P90L is highly conserved with extremely low population frequencies and no homozygotes in ExAC Browser. Its clinical significance remains uncertain in ClinVar. The tri-allelic mutations in *RNASEH2B* may cause a synergistic pathogenic effect since neither heterozygous nor homozygous variants alone can account for her skin and neurological manifestations.

This patient has demonstrated a later onset of AGS with average intelligence, presenting with chilblains, cerebral atrophy, white matter abnormalities, intracranial calcification, and over-expression of Interferon-stimulated genes. Besides, this patient has persistent systemic inflammation and chronic renal dysfunction, which are uncommon in AGS (Table 2). Mixed connective tissue diseases have been excluded by the systemic evaluation. Systemic juvenile idiopathic arthritis (SoJIA), and later onset chronic infantile neurologic, cutaneous, and arthritis (CINCA) syndrome were once suspected. Different from the clinical manifestations of this patient, chilblains and intracranial calcification are not present in SoJIA or CINCA; leukocytosis, destructive arthritis, or macrophage activation syndrome (MAS) are noted in SoJIA [4–6]; visual impairment, sensor neural deafness or progressive chronic meningitis have been commonly reported in CINCA [5]. Chronic kidney disease due to amyloidosis has been rarely reported in SoJIA, which is common in CINCA (Table 2) [8].

**Table 2:** Comparison of clinical features among AGS, soJIA, and CINCA

Clinical features	Our Patient	Other AGS [2, 3]	soJIA [4–6]	CINCA [7, 8]
Onset within the first year of life	no	common	rare	common
Fever	frequently often	rare	common	common
Preserved or normal intelligence	yes	less common	yes	less common
Mental retardation	no	common	rare	common
Joint swelling	yes	rare	common	common
Arthralgia	yes	rare	common	common
Destructive arthritis	no	rare	common	common
Chilblains	yes	common	NR	rare
Urticarial rash	no	rare	less common	common
Salmon-pink rash	no	rare	common	less common
Conjunctivitis	no	none	less common	common
Visual damage	no	less common	rare	common
Sensor neural deafness	no	rare	NR	common
Progressive chronic meningitis	no	rare	NR	common
Auto-inflammatory manifestations	yes	less common	common	common
Auto-immune manifestations	no	common	rare	none
Severe intra-uterine growth retardation	no	common	NR	rare
Microcephaly	no	common	NR	rare
Psychomotor retarded	not obvious	common	rare	common
Feeding difficulties	no	common	NR	less common
Growth retardation	yes	common	less common	common
Hepatosplenomegaly	no	common	common	common
Cerebral atrophy	milder	common	NR	common
White matter abnormalities	yes	common	NR	less common
Intracranial calcification	yes	common	NR	none
Chronic kidney disease	yes	none	rare	common
Renal amyloidosis	not yet	none	rare	common
Leukocytosis	no	rare	common	common
C-reactive protein	significantly elevated	rare	significantly elevated	significantly elevated
Erythrocyte sedimentation	significantly elevated	less common	significantly elevated	significantly elevated
Ferritin	normal	normal	significantly elevated in MAS	significantly elevated in MAS
Triglycerides	normal	normal	elevated in MAS	elevated in MAS

*AGS* Aicardi-Goutières, *SoJIA* Systemic juvenile idiopathic arthritis, *CINCA* onset chronic infantile neurologic, cutaneous, and arthritis syndrome, *NR* not reported, *MAS* macrophage activation syndrome

Renal involvement has been described in a case with a gain-of-function mutation in *IFIH1* [9]. Renal dysfunction caused by thrombotic microangiopathy has been reported in a case with C-terminal frame-shift mutation in *TREX1* [10]. Renal biopsy in our patient revealed glomerular sclerosis and tubular injury without amyloidosis. RNASEH2B is moderately expressed in the kidney. Pathogenic variations in *RNASEH2B* might impair the normal function of kidney directly, or secondary to chronic inflammation. Human IFN-alpha is filtrated by the kidney, primarily reabsorbed, most probably catabolized within the tubular epithelium, and excreted in negligible amounts with the urine [11]. A fairly high IFN-a level within the tubular epithelium due to a persistently elevated IFN-a level in plasma might amplify the activation of the interferon pathway, leading to the infiltration of lymphocytes and mononuclear cells, and local chronic inflammation. Further investigations will help to explore the distinct pathogenesis underlying chronic renal dysfunction in the *RNASEH2B* defect.

IL-6 is one of the downstream effector cytokines in the IFN signaling pathway. IL-6 blockade has good efficacy in a patient with a cerebral vasculopathy due to a homozygous *SAMHD1* mutation [12]. Tocilizumab has partial efficacy in this patient, leading to a reduction of acute-phase reactants. However, it has failed to improve the chronic renal tubular disease. Further clinical trials are required to clarify the efficacy of tocilizumab in AGS.

IFN-α and IFN-β act on type I receptors (IFNAR1/2) to activate the Janus kinase (JAK)-signal transducers and activators of the transcription (STAT) pathway. JAK inhibitors have good efficacy in patients with some type I interferonopathies, including STING-associated vasculopathy, infantile-onset (SAVI), and proteasome-associated autoinflammatory syndrome (PRAAS) [13–16]. Sustained elevated IFN-α and IFN-β levels are common in AGS. JAK inhibitors can theoretically help to reduce the autoinflammation in AGS. Ruxolitinib has reduced neuroinflammation in a patient with a heterozygous mutation in *IFIH1* [17]. Baricitinib could alleviate chilblain lesions in a patient with AGS5 [18]. Tofacitinib ameliorated aortic valve calcification in a patient with Singleton-Merten syndrome (SMS) [19]. Ruxolitinib led to an improvement of psychomotor delay with a reduction in dystonic movements in two patients with AGS2 [20]. However, ruxolitinib failed to prevent the onset of clinical signs in a patient with *RNASEH2B* mutation [21]. Tofacitinib demonstrated a partial response in this patient, failing to ameliorate autoinflammation and chronic kidney disease completely. Therefore, based on limited case reports, the efficacy of JAK inhibitors in AGS remains uncertain. The currently ongoing trial conducted at the Children’s Hospital of Philadelphia (ClinicalTrials.gov number, NCT03921554) will help to explore the efficacy and safety of baricitinib in AGS and AGS-related interferonopathies.

## Conclusions

We have described a patient with both homozygous and heterozygous variants in *RNASEH2B*, revealing a possible synergistic pathogenic effect among variants in the same gene. Her systemic autoinflammation and chronic kidney disease will expand the clinical phenotype spectrum of this syndrome. The pathogenesis underlying chronic renal dysfunction in this patient remains poorly understood. The efficacy of tocilizumab and JAK inhibitors in AGS remains uncertain, and further clinical researches are needed.

## Supplementary Information

**Additional file 1.**

**Additional file 2.**

## Data Availability

Clinical datasets were collected from medical records of the participated patient in Shenzhen Children’s hospital.

## Abbreviations

AGS: Aicardi-Goutières
WES: Whole exome sequencing
PBMCs: Peripheral blood mononuclear cells
cGAMP: cyclic guanosine monophosphate-adenosine monophosphate
IQ: Intelligence Quotient
MRI: Magnetic resonance imaging
RF: Rheumatoid factor
ANA: Anti-nuclear antibody
ANCA: Anti-neutrophil cytoplasmic antibody
TNFα: Tumor necrosis factor-α
IFN-γ: Interferon-γ
CRP: C-reactive protein
ESR: Erythrocyte sedimentation rate
SAA: Serum amyloid A
SLE: Systemic lupus erythematosus
SoJIA: Systemic juvenile idiopathic arthritis
CINCA: Onset chronic infantile neurologic, cutaneous, and arthritis syndrome
MAS: Macrophage activation syndrome
JAK: Janus kinase
SAVI: STING-associated vasculopathy, infantile-onset
PRAAS: Proteasome-associated autoinflammatory syndrome
SMS: Singleton-Merten syndrome
