# Supplementary material for: Systemic inflammation and chronic kidney disease in a patient due to the RNASEH2B defect
Source: Pediatr Rheumatol Online J. 2021 Jan 22;19:9. doi: 10.1186/s12969-021-00497-2 (PMC7821736; doi:10.1186/s12969-021-00497-2)
Supplement: Supplementary file 1 — Additional file 1. [file 12969_2021_497_MOESM1_ESM.docx]

**Primers for Real-time PCR**

Actin: TTGCCGACAGGATGCAGAAG (F) and TGGACAGCGAGGCCAGGATG (R);

IFNβ1: AGCAGTTCCAGAAGGAGGAC (F) and GCCAGGAGGTTCTCAACAAT (R);

IFNα4: GAATCTCTCATTTCTCCTGCC (F) and TGTGCTGAAGAGATTGAAGG (R);

IFI44: GGTGGGCACTAATACAACTGG (F) and AGTCACACAGAATAAACGGCAG (R);

IFI27: CTCTGCTCTCACCTCATCAG (F) and CCACAACTCCTCCAATCAC (R);

IFIT1: AGCCTGGCTAAGCAAAACCC (F) and TTCATCGTCATCAATGGATAACTC (R);

IFIT2: TTTATTGGTGGCAGAAGAGG (F) and TAGTTGCCGTAGGCTGCTCT (R);

IFIT3: AACAGCAGAGACACAGAGGG (F) and AAGTTCCAGGTGAAATGGC (R);

ISG15: ACTCATCTTTGCCAGTACAG (F) and CTTCAGCTCTGACACCGACAT (R);

OAS1: GTGTGTCCAAGGTGGTAAAG (F) and CTCTCCCCGGCGATTTAACT (R);

SIGLEC1: TTCCTACAACTTCCGCTTCG (F) and CACCCTGGGCTCCTCTGTT (R) ;

IRF9: CAGTTGCTGCCACCAGGAAT (F) and ATTGAGGGAGTCCTGGAGCAC (R);

RASD2: GCAGGCTGTCCATCCTCAC (F) and TCTGCTTCTGAAGGCGCTTG (R)
